# Association and incremental predictive value of preoperative AISI and CALLY for postoperative pulmonary complications after McKeown esophagectomy following neoadjuvant chemoimmunotherapy

**DOI:** 10.3389/fimmu.2026.1642365

**Published:** 2026-04-15

**Authors:** Ruihua Fan, Rong Yao, Jianqiang Zhao, Wenze Tian, Zhiyun Xu

**Affiliations:** 1 Department of Oncology, The Affiliated Huai’an No. 1 People’s Hospital of Nanjing Medical University, Huai’an, China; 2 Department of Thoracic Surgery, The Affiliated Huai’an No. 1 People’s Hospital of Nanjing Medical University, Huai’an, China

**Keywords:** AISI, CALLY index, esophageal squamous cell carcinoma, McKeown esophagectomy, neoadjuvant chemoimmunotherapy, postoperative pulmonary complications

## Abstract

**Background:**

Postoperative pulmonary complications (PPCs) remain a major source of morbidity after McKeown esophagectomy for esophageal squamous cell carcinoma (ESCC), particularly in patients receiving neoadjuvant chemoimmunotherapy (nICT). Readily available preoperative biomarkers may improve risk stratification. This study evaluated the predictive value of the aggregate index of systemic inflammation (AISI), the C-reactive protein-albumin-lymphocyte (CALLY) index, and their combined use for PPCs after McKeown esophagectomy following nICT.

**Methods:**

We retrospectively analyzed 412 consecutive ESCC patients who underwent McKeown esophagectomy after nICT between January 2019 and December 2025. The primary endpoint was PPCs within 30 days after surgery. Univariable and multivariable logistic regression analyses were used to examine associations between preoperative biomarkers and PPCs. A combined AISI-CALLY model was constructed using binary logistic regression, and its predictive performance was assessed by receiver operating characteristic (ROC) analysis, DeLong testing, calibration analysis, and decision curve analysis. Propensity score matching (PSM) was performed as a sensitivity analysis.

**Results:**

PPCs occurred in 157 of 412 patients (38.1%). In the fully adjusted model, both AISI and CALLY remained independently associated with PPCs. Higher AISI was associated with increased PPC risk (adjusted odds ratio [aOR] 1.195 per 100-unit increase, 95% CI 1.081-1.321, *P* < 0.001), whereas higher CALLY was associated with lower risk (aOR 0.926 per 1-unit increase, 95% CI 0.884-0.970, *P* = 0.001). The biomarker-only AISI-CALLY model achieved an AUC of 0.689, compared with 0.650 for AISI alone and 0.665 for CALLY alone. The final integrated model incorporating clinical variables, AISI, and CALLY showed the best discrimination (AUC 0.712, 95% CI 0.658-0.762) and provided greater net benefit on decision curve analysis. In the matched cohort, both biomarkers remained independently associated with PPCs, although discrimination was attenuated.

**Conclusion:**

Preoperative AISI and CALLY were independently and complementarily associated with PPCs after McKeown esophagectomy following nICT. Their combined use provided only modest incremental predictive value and may serve as an accessible adjunct, rather than a stand-alone tool, for preoperative PPC risk stratification.

## 1 Introduction

Esophageal cancer remains a major global health burden, and esophageal squamous cell carcinoma (ESCC) continues to account for a substantial proportion of cases in Asian populations (1, 2). Multimodal treatment has become standard for locally advanced disease, but curative management still depends heavily on esophagectomy. In this study, postoperative pulmonary complications (PPCs) remain among the most frequent and clinically consequential adverse events after surgery because they delay recovery, prolong hospitalization, increase perioperative morbidity, and may interfere with subsequent oncologic care (3, 4). Recent review-level evidence also indicates that esophagectomy continues to carry a high postoperative complication burden despite advances in perioperative management and surgical technique (5).

The perioperative landscape of ESCC has changed substantially in recent years (6). Neoadjuvant therapy is now an essential component of treatment for resectable locally advanced disease, and neoadjuvant chemoimmunotherapy (nICT) is being increasingly adopted because of its promising pathological activity and encouraging short-term outcomes (7). At the same time, this treatment paradigm may alter perioperative risk in ways that are not fully captured by conventional clinical assessment (8). Cytotoxic chemotherapy can aggravate catabolic stress, myelosuppression, and lymphocyte depletion, whereas immune checkpoint blockade can reshape host immune homeostasis and, in some patients, induce pulmonary or systemic inflammatory toxicity (9). These treatment-related perturbations may become especially relevant before major thoracic surgery, where postoperative recovery depends not only on technical success but also on the patient’s inflammatory burden, nutritional reserve, immune competence, and cardiopulmonary resilience (10).

This concern is particularly important in patients undergoing McKeown esophagectomy (11). Although this procedure remains widely used for ESCC because it enables radical resection and extensive lymphadenectomy, it also involves substantial thoracic manipulation, single-lung ventilation, and postoperative airway vulnerability, all of which contribute to respiratory morbidity (12). In the present study, PPCs were defined as a 30-day composite endpoint including pneumonia, pleural effusion, atelectasis, respiratory failure, reintubation, and prolonged mechanical ventilation, reflecting the clinically relevant spectrum of postoperative respiratory events in this setting (13). Because these complications arise from the interaction between surgical stress and host biological vulnerability, preoperative identification of patients at increased risk is essential for perioperative planning, respiratory optimization, and more individualized postoperative surveillance (14).

Traditional risk stratification for PPCs after esophagectomy has mainly relied on demographic characteristics, comorbidities, pulmonary function, anesthetic risk classification, and procedural variables (3). These factors remain clinically important, but they do not fully reflect the biological state of patients who have already undergone systemic neoadjuvant treatment. In recent years, increasing attention has been directed toward blood-based composite biomarkers because they are inexpensive, objective, and readily available in routine practice. Among them, the aggregate index of systemic inflammation (AISI), calculated from neutrophil, platelet, monocyte, and lymphocyte counts, has been proposed as a marker of systemic inflammatory burden (15, 16). The C-reactive protein-albumin-lymphocyte (CALLY) index integrates acute-phase inflammation, nutritional status, and immune competence into a single measure (17). In our study design, these biomarkers were therefore selected to represent two biologically related but conceptually distinct dimensions of preoperative risk: systemic inflammation for AISI and integrated inflammation-nutrition-immune status for CALLY.

The rationale for studying these two markers together is biologically plausible. Elevated systemic inflammation may impair pulmonary microcirculation, amplify perioperative tissue injury, and weaken host defense, whereas poor nutritional and immune status may reduce tolerance to operative stress and increase susceptibility to postoperative infection (18). Existing evidence has already suggested that a lower preoperative CALLY index is associated with poorer oncologic outcomes in esophageal cancer and may also be linked to postoperative infectious complications (19). At the same time, inflammation-based indices such as AISI are increasingly being explored across cancer populations as accessible markers of host vulnerability (20). However, the perioperative relevance of these biomarkers may differ according to treatment context, operative approach, and endpoint definition, which makes disease- and setting-specific evaluation necessary.

Despite this emerging interest, several important gaps remain. First, evidence specifically addressing PPC risk after McKeown esophagectomy in the era of nICT is still limited. Much of the existing literature has focused on long-term prognosis, general postoperative morbidity, or single complications such as pneumonia rather than a broader composite PPC endpoint after contemporary multimodal therapy. Second, although AISI and CALLY both appear biologically relevant, their independent and complementary roles in this setting have not been clearly defined. In our cohort, the two biomarkers were only moderately correlated and showed no substantial multicollinearity, supporting the premise that they may capture nonredundant information rather than serve as interchangeable surrogates. Third, whether these readily available biomarkers can provide incremental predictive value beyond standard clinical variables remains uncertain, yet this is the question most relevant to real-world perioperative decision-making.

Against this background, we conducted a retrospective cohort study of consecutive patients with ESCC who underwent McKeown esophagectomy after nICT to evaluate the association and incremental predictive value of preoperative AISI and CALLY for PPCs within 30 days after surgery. We further examined whether these two biomarkers could be jointly incorporated into a combined model and whether their addition improved predictive performance beyond a baseline clinical model. Because AISI and CALLY reflect overlapping yet distinct aspects of host biology, we hypothesized that they would show independent and complementary associations with PPCs and could serve as accessible adjuncts for preoperative risk stratification in this setting.

## 2 Methods

### 2.1 Study design and patient selection

This retrospective cohort study included consecutive patients with esophageal squamous cell carcinoma (ESCC) who underwent McKeown esophagectomy after neoadjuvant chemoimmunotherapy (nICT) at the Affiliated Huai’an No. 1 People’s Hospital of Nanjing Medical University between January 2019 and December 2025. The study was conducted in accordance with the Declaration of Helsinki and was approved by the Institutional Ethics Committee of Nanjing Medical University (Approval No. KY-2024-374-01), which waived the requirement for informed consent because of the retrospective design. Eligible patients were required to meet the following criteria: (1) histologically confirmed ESCC; (2) receipt of nICT before surgery; (3) subsequent McKeown esophagectomy with curative intent; (4) availability of complete preoperative laboratory data for calculation of the aggregate index of systemic inflammation (AISI) and the C-reactive protein-albumin-lymphocyte (CALLY) index; and (5) complete postoperative follow-up data for assessment of postoperative pulmonary complications (PPCs) within 30 days after surgery. Patients were excluded if they had: (1) pre-existing active pulmonary infection or severe chronic pulmonary disease that could substantially affect postoperative pulmonary outcomes; (2) previous thoracic surgery; (3) prior or coexisting malignancies; (4) hematologic or immune disorders that could influence inflammatory biomarker measurements; or (5) incomplete clinicopathological or laboratory records. After application of the inclusion and exclusion criteria, 412 patients were included in the final analytic cohort.

### 2.2 Data collection and variable definition

Clinical data were retrospectively extracted from the institutional electronic medical records and surgical database. The collected variables included demographic characteristics, comorbidities, preoperative functional status, pulmonary and cardiac function, tumor-related features, perioperative variables, laboratory biomarkers, and postoperative outcomes. Demographic and baseline clinical variables included age, sex, body mass index (BMI), smoking history, and drinking history. Comorbidity-related variables included hypertension, diabetes mellitus, coronary heart disease (CHD), and chronic obstructive pulmonary disease (COPD). Preoperative physical status was assessed using the American Society of Anesthesiologists (ASA) classification and the Eastern Cooperative Oncology Group (ECOG) performance status. Preoperative cardiopulmonary function variables included forced expiratory volume in 1 second (FEV1), the FEV1/forced vital capacity (FVC) ratio, and left ventricular ejection fraction (LVEF). Tumor-related variables included tumor location, tumor grade, pathological T stage (ypT), pathological N stage (ypN), and pathological TNM stage (ypTNM) after neoadjuvant treatment. Perioperative variables included the interval from nICT to surgery, operative duration, single-lung ventilation time, intraoperative blood loss, and surgical approach. Preoperative laboratory variables were collected from routine blood tests performed before surgery and included serum albumin, C-reactive protein (CRP), neutrophil count, lymphocyte count, monocyte count, and platelet count.

### 2.3 Definition of biomarkers: AISI and CALLY

Preoperative AISI and CALLY were calculated using routinely available laboratory parameters obtained from peripheral blood samples collected before surgery. AISI was calculated as neutrophil count × platelet count × monocyte count/lymphocyte count (15). CALLY was calculated as serum albumin × lymphocyte count/C-reactive protein. AISI was used as a composite marker reflecting systemic inflammatory burden, whereas CALLY was used as a composite index integrating inflammatory, nutritional, and immune status (21). Both biomarkers were analyzed primarily as continuous variables. To improve clinical interpretability in regression analyses, AISI was modeled per 100-unit increase, whereas CALLY was modeled per 1-unit increase.

### 2.4 Study endpoint

The primary endpoint of this study was postoperative pulmonary complications (PPCs) occurring within 30 days after surgery. PPCs were treated as a composite endpoint comprising clinically relevant respiratory complications arising during the postoperative period (22, 23). The definition of PPCs and the identification of individual pulmonary events were based on predefined clinical criteria with reference to established perioperative pulmonary complication definitions reported in the literature. The composite PPC endpoint included postoperative pneumonia, pleural effusion, atelectasis, respiratory failure, reintubation, and prolonged mechanical ventilation. These events were identified through review of postoperative clinical records, imaging findings, respiratory support requirements, and discharge documentation. If more than one pulmonary complication occurred in the same patient, the patient was considered to have reached the composite PPC endpoint. For the primary analysis, patients were classified into PPC and non-PPC groups according to whether any PPC occurred within 30 days after surgery. Individual pulmonary complications were additionally recorded for descriptive and exploratory analyses.

### 2.5 Statistical analysis

#### 2.5.1 Descriptive analysis and baseline comparison

Continuous variables were expressed as mean ± standard deviation (SD) or median (interquartile range [IQR]), as appropriate according to data distribution, whereas categorical variables were presented as number (percentage). Normality of continuous variables was assessed using the Shapiro-Wilk test together with visual inspection of distribution patterns. For baseline comparisons, patients were divided into PPC and non-PPC groups according to the occurrence of PPCs within 30 days after surgery. Between-group comparisons were performed using the Student’s t test for normally distributed continuous variables and the Mann-Whitney U test for non-normally distributed continuous variables. Categorical variables were compared using the chi-square test or Fisher’s exact test, as appropriate.

#### 2.5.2 Correlation and collinearity assessment

The distributions of AISI and CALLY were first examined descriptively. The association between the two biomarkers was assessed using Spearman’s rank correlation coefficient because of their non-normal distribution. To determine whether AISI and CALLY could be simultaneously entered into the same regression model, multicollinearity was evaluated using variance inflation factors (VIFs) and tolerance values. A VIF <5 and a tolerance >0.2 were considered indicative of the absence of substantial multicollinearity.

#### 2.5.3 Univariable logistic regression analysis

Univariable logistic regression analyses were performed to evaluate the association between each candidate variable and the risk of PPCs. Odds ratios (ORs) with 95% confidence intervals (CIs) were calculated for demographic characteristics, comorbidities, preoperative functional variables, tumor-related factors, perioperative variables, and biomarker variables. Continuous variables were entered into the models as continuous terms. AISI was modeled per 100-unit increase, whereas CALLY was modeled per 1-unit increase. For categorical variables with more than two levels, the lowest category was used as the reference group.

#### 2.5.4 Multivariable logistic regression analysis

Multivariable logistic regression models were constructed to identify independent predictors of PPCs while adjusting for potential confounding factors. A stepwise modeling strategy was used. Model 1 consisted of the baseline clinical model, which included clinically relevant covariates selected on the basis of prior clinical knowledge and perioperative relevance. Model 2 included the clinical model plus AISI. Model 3 included the clinical model plus CALLY. Model 4 included the clinical model plus both AISI and CALLY. Adjusted odds ratios (aORs) with 95% confidence intervals (CIs) were reported for all multivariable models.

#### 2.5.5 Construction of the combined AISI-CALLY model

To evaluate the joint predictive value of AISI and CALLY, a biomarker-based combined AISI-CALLY model was constructed using binary logistic regression, with PPCs as the dependent variable and AISI and CALLY as the predictors. The predicted probabilities derived from this model were used as continuous prediction scores in subsequent model performance analyses. In addition to the biomarker-only combined model, an integrated final model was also evaluated by incorporating both AISI and CALLY into the baseline clinical model.

#### 2.5.6 Discrimination analysis

Model discrimination was assessed using receiver operating characteristic (ROC) curve analysis, and the area under the ROC curve (AUC) was calculated with corresponding 95% CIs. ROC analyses were performed for AISI alone, CALLY alone, the combined AISI-CALLY model, the baseline clinical model, and the integrated models incorporating AISI and/or CALLY. Comparisons between AUCs were performed using the DeLong test. In addition, model performance metrics including optimal cutoff value, sensitivity, specificity, positive predictive value (PPV), negative predictive value (NPV), accuracy, and Youden index were calculated. The optimal cutoff value for each model was determined using the Youden index.

#### 2.5.7 Calibration analysis

Calibration of the final integrated model was evaluated using a calibration curve comparing predicted and observed risks of PPCs. Model calibration was further assessed using the Brier score and the Hosmer-Lemeshow goodness-of-fit test.

#### 2.5.8 Clinical utility analysis

The clinical usefulness of the prediction models was evaluated using decision curve analysis (DCA). Net benefit was calculated across a range of threshold probabilities for the baseline clinical model, the combined AISI-CALLY model, and the final integrated model. The “treat all” and “treat none” strategies were included as reference standards.

#### 2.5.9 Propensity score matching analysis

To further assess the robustness of the associations between AISI, CALLY, and PPCs, propensity score matching (PSM) was performed as a sensitivity analysis. Propensity scores were estimated using a logistic regression model based on baseline clinicopathological and perioperative variables that could potentially influence the occurrence of PPCs. Patients with PPCs were matched 1:1 to patients without PPCs using nearest-neighbor matching without replacement, with a caliper width of 0.2 standard deviations of the logit of the propensity score. Covariate balance before and after matching was assessed using standardized mean differences (SMDs), and an absolute SMD <0.1 was considered indicative of acceptable balance. After matching, baseline characteristics were re-evaluated, and multivariable logistic regression and ROC analyses were repeated in the matched cohort.

#### 2.5.10 Software and statistical significance

All statistical analyses were performed using R software (version 4.2.2) and Python (version 3.12). A two-sided P value <0.05 was considered statistically significant.

## 3 Results

### 3.1 Patient selection and baseline characteristics

**Figure 1:**
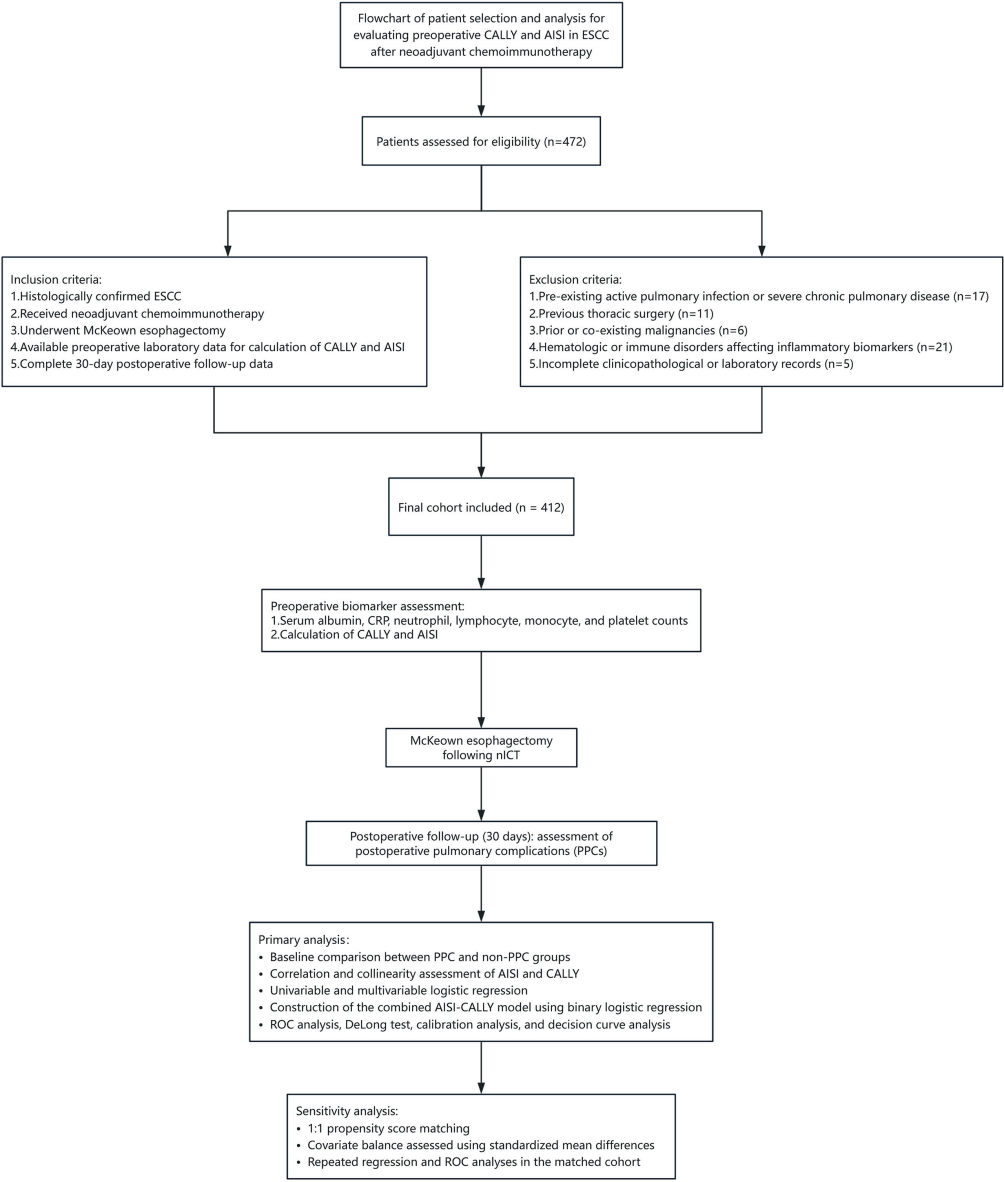
Study flowchart and analytical workflow. Flowchart of patient selection and overall analytical workflow. A total of 472 patients were screened, and 412 patients with esophageal squamous cell carcinoma who underwent McKeown esophagectomy after neoadjuvant chemoimmunotherapy were included in the final analytic cohort. The primary endpoint was postoperative pulmonary complications (PPCs) within 30 days after surgery. The analytical workflow included baseline comparison, assessment of the distribution, correlation, and collinearity of AISI and CALLY, univariable and multivariable logistic regression analyses, evaluation of discrimination, calibration, and clinical utility, and propensity score matching as a sensitivity analysis.

A total of 472 patients were initially screened for eligibility. After application of the predefined inclusion and exclusion criteria, 412 patients with esophageal squamous cell carcinoma (ESCC) who underwent McKeown esophagectomy after neoadjuvant chemoimmunotherapy (nICT) were included in the final analytic cohort (**Figure 1**). Postoperative pulmonary complications (PPCs) occurred in 157 patients (38.1%), whereas 255 patients (61.9%) did not develop PPCs. Among individual pulmonary complications, postoperative pneumonia was the most frequent event, occurring in 108 patients (26.2%), followed by atelectasis in 36 patients (8.7%) and pleural effusion in 31 patients (7.5%). Respiratory failure, prolonged mechanical ventilation, and reintubation were observed less frequently. Baseline clinicopathological and perioperative characteristics according to PPC status are summarized in **Table 1**. Compared with patients without PPCs, those who developed PPCs had significantly longer operative duration, higher preoperative C-reactive protein (CRP) levels, higher aggregate index of systemic inflammation (AISI) values, and lower C-reactive protein-albumin-lymphocyte (CALLY) index values. Most other demographic, comorbidity-related, pulmonary function, tumor-related, and perioperative variables were generally comparable between the two groups.

**Table 1:** Baseline characteristics according to PPC status.

Variable	Overall (n=412)	No PPC (n=255)	PPC (n=157)	P value
Age, years	68.21 ± 5.11	67.89 ± 5.15	68.71 ± 5.02	0.112
BMI, kg/m²	22.87 ± 2.39	22.81 ± 2.26	22.98 ± 2.59	0.504
FEV1, L	2.17 ± 0.42	2.14 ± 0.42	2.21 ± 0.41	0.105
FEV1/FVC, %	103.91 ± 12.15	104.77 ± 11.98	102.51 ± 12.33	0.069
LVEF, %	66.10 ± 2.30	66.24 ± 2.26	65.87 ± 2.35	0.111
nICT-to-surgery interval, days	30.63 ± 7.39	30.75 ± 7.26	30.43 ± 7.61	0.667
Operative duration, min	275.10 ± 38.24	272.08 ± 37.77	280.00 ± 38.60	0.042
Single-lung ventilation time, min	146.27 ± 28.54	145.02 ± 28.26	148.30 ± 28.97	0.260
Blood loss, mL	130.74 ± 44.71	128.71 ± 44.51	134.04 ± 44.99	0.242
Albumin, g/L	39.65 ± 3.61	39.65 ± 3.57	39.64 ± 3.68	0.971
CRP, mg/L	7.17 (4.78–10.09)	6.51 (4.45–9.16)	8.32 (5.85–11.66)	<0.001
CALLY index	7.48 (4.91–11.81)	8.68 (5.53–12.80)	5.72 (4.28–8.06)	<0.001
AISI	293.88 (199.07–466.47)	260.06 (174.52–409.29)	385.94 (228.00–548.28)	<0.001
Male sex, n (%)	235 (57.0%)	150 (58.8%)	85 (54.1%)	0.406
Smoking history, n (%)	147 (35.7%)	83 (32.5%)	64 (40.8%)	0.113
Drinking history, n (%)	110 (26.7%)	64 (25.1%)	46 (29.3%)	0.411
Hypertension, n (%)	183 (44.4%)	114 (44.7%)	69 (43.9%)	0.962
Diabetes, n (%)	69 (16.7%)	42 (16.5%)	27 (17.2%)	0.955
CHD, n (%)	142 (34.5%)	82 (32.2%)	60 (38.2%)	0.250
COPD, n (%)	31 (7.5%)	15 (5.9%)	16 (10.2%)	0.156
Minimally invasive approach, n (%)	316 (76.7%)	196 (76.9%)	120 (76.4%)	1.000
ASA class				0.629
ASA 1, n (%)	111 (26.9%)	67 (26.3%)	44 (28.0%)	
ASA 2, n (%)	277 (67.2%)	171 (67.1%)	106 (67.5%)	
ASA 3, n (%)	24 (5.8%)	17 (6.7%)	7 (4.5%)	
ECOG				0.547
ECOG 0, n (%)	216 (52.4%)	133 (52.2%)	83 (52.9%)	
ECOG 1, n (%)	165 (40.0%)	100 (39.2%)	65 (41.4%)	
ECOG 2, n (%)	31 (7.5%)	22 (8.6%)	9 (5.7%)	
Tumor location				0.135
Upper, n (%)	98 (23.8%)	64 (25.1%)	34 (21.7%)	
Middle, n (%)	208 (50.5%)	134 (52.5%)	74 (47.1%)	
Low, n (%)	106 (25.7%)	57 (22.4%)	49 (31.2%)	
Grade				0.206
Well, n (%)	235 (57.0%)	154 (60.4%)	81 (51.6%)	
Moderate, n (%)	145 (35.2%)	82 (32.2%)	63 (40.1%)	
Poor, n (%)	32 (7.8%)	19 (7.5%)	13 (8.3%)	
ypT stage				0.370
ypT0, n (%)	131 (31.8%)	74 (29.0%)	57 (36.3%)	
ypT1, n (%)	178 (43.2%)	118 (46.3%)	60 (38.2%)	
ypT2, n (%)	84 (20.4%)	51 (20.0%)	33 (21.0%)	
ypT3, n (%)	19 (4.6%)	12 (4.7%)	7 (4.5%)	
ypN stage				0.286
ypN0, n (%)	326 (79.1%)	203 (79.6%)	123 (78.3%)	
ypN1, n (%)	63 (15.3%)	38 (14.9%)	25 (15.9%)	
ypN2, n (%)	15 (3.6%)	7 (2.7%)	8 (5.1%)	
ypN3, n (%)	8 (1.9%)	7 (2.7%)	1 (0.6%)	
ypTNM stage				0.309
0, n (%)	110 (26.7%)	63 (24.7%)	47 (29.9%)	
I, n (%)	202 (49.0%)	129 (50.6%)	73 (46.5%)	
II, n (%)	92 (22.3%)	56 (22.0%)	36 (22.9%)	
III, n (%)	8 (1.9%)	7 (2.7%)	1 (0.6%)	

Data are presented as mean ± standard deviation (SD), median (interquartile range [IQR]), or number (percentage), as appropriate. P values were calculated using the Student’s t test, Mann–Whitney U test, chi-square test, or Fisher’s exact test, as appropriate. PPCs, postoperative pulmonary complications; BMI, body mass index; FEV1, forced expiratory volume in 1 second; FVC, forced vital capacity; LVEF, left ventricular ejection fraction; CRP, C-reactive protein; AISI, aggregate index of systemic inflammation; CALLY, C-reactive protein-albumin-lymphocyte index; CHD, coronary heart disease; COPD, chronic obstructive pulmonary disease; ECOG, Eastern Cooperative Oncology Group.

### 3.2 Distribution, correlation, and collinearity of AISI and CALLY

The distributions of preoperative AISI and CALLY in the overall cohort are shown in **Supplementary Figure 1**. AISI demonstrated a right-skewed distribution with a relatively wide range of values, whereas CALLY showed a comparatively narrower distribution. Spearman correlation analysis showed that AISI and CALLY were moderately and inversely correlated (Spearman’s ρ = -0.391, P < 0.001), indicating that higher systemic inflammatory burden tended to be associated with lower inflammation-nutrition-immune composite status. However, the magnitude of the correlation was not sufficiently strong to suggest that the two biomarkers were interchangeable. Collinearity diagnostics demonstrated no substantial multicollinearity between AISI and CALLY. The variance inflation factors were 1.11 for both biomarkers, and the corresponding tolerance values were 0.904. These findings supported the simultaneous inclusion of AISI and CALLY in the same multivariable and combined prediction models.

### 3.3 Univariable and multivariable analyses for PPCs

The results of the univariable logistic regression analyses are presented in **Table 2**. In univariable analysis, longer operative duration, higher preoperative CRP, higher AISI, and lower CALLY were significantly associated with PPCs. Notably, each 100-unit increase in AISI was associated with increased odds of PPCs, whereas each 1-unit increase in CALLY was associated with reduced odds of PPCs. Multivariable logistic regression analyses are summarized in **Table 3**. In the baseline clinical model, lower FEV1/FVC (aOR 0.981, 95% CI 0.964–0.997, P = 0.024) and longer operative duration (aOR 1.007, 95% CI 1.001–1.012, P = 0.019) were independently associated with PPCs. After addition of AISI, AISI remained an independent predictor of PPCs (aOR 1.257 per 100 units, 95% CI 1.141–1.384, P < 0.001). Likewise, after addition of CALLY, CALLY also remained independently associated with PPCs (aOR 0.903 per unit, 95% CI 0.862–0.945, P < 0.001). Importantly, in the fully adjusted model including both biomarkers, AISI and CALLY both retained statistical significance. AISI remained associated with a higher risk of PPCs (aOR 1.195 per 100 units, 95% CI 1.081–1.321, P < 0.001), whereas CALLY remained associated with a lower risk of PPCs (aOR 0.926 per unit, 95% CI 0.884–0.970, P = 0.001). These findings indicate that AISI and CALLY provided independent and complementary prognostic information.

**Table 2:** Univariable logistic regression analysis for PPCs.

Variable	Comparison	OR (95% CI)	P value
Demographic and clinical characteristics			
Age (years)	Per 1-unit increase	1.032 (0.992–1.073)	0.115
Sex	Male vs Female	0.826 (0.553–1.234)	0.351
BMI (kg/m²)	Per 1-unit increase	1.030 (0.948–1.119)	0.490
Smoking history	Yes vs No	1.426 (0.944–2.154)	0.092
Drinking history	Yes vs No	1.237 (0.792–1.930)	0.350
Hypertension	Yes vs No	0.970 (0.650–1.447)	0.881
Diabetes	Yes vs No	1.053 (0.620–1.790)	0.848
CHD	Yes vs No	1.305 (0.861–1.977)	0.209
COPD	Yes vs No	1.816 (0.871–3.784)	0.111
ASA	2 vs 1	0.944 (0.601–1.482)	0.802
	3 vs 1	0.627 (0.240–1.636)	0.340
ECOG	1 vs 0	1.042 (0.687–1.578)	0.848
	2 vs 0	1.156 (0.288–1.492)	0.314
Pulmonary and cardiac function			
FEV1 (L)	Per 1-unit increase	1.481 (0.917–2.391)	0.108
FEV1/FVC (%)	Per 1-unit increase	0.985 (0.968–1.001)	0.067
LVEF (%)	Per 1-unit increase	0.931 (0.853–1.016)	0.108
Tumor and pathological factors			
Tumor location	Middle vs Upper	1.040 (0.628–1.720)	0.880
	Low vs Upper	1.618 (0.920–2.846)	0.095
Tumor grade	Moderate vs Well	1.461 (0.955–2.233)	0.080
	Poor vs Well	1.301 (0.611–2.768)	0.495
ypT stage	T1 vs T0	1.060 (0.415–1.251)	0.080
	T2 vs T0	1.140 (0.481–1.467)	0.540
	T3 vs T0	1.707 (0.280–2.046)	0.584
ypN stage	N1 vs N0	1.086 (0.625–1.886)	0.770
	N2 vs N0	1.886 (0.667–5.330)	0.231
	N3 vs N0	1.236 (0.029–1.939)	0.179
ypTNM stage	I vs 0	1.059 (0.472–1.219)	0.254
	II vs 0	1.062 (0.490–1.514)	0.605
	III vs 0	1.091 (0.023–1.610)	0.128
Perioperative factors			
nICT-to-surgery interval (days)	Per 1-unit increase	0.994 (0.968–1.021)	0.663
Operative duration (min)	Per 1-unit increase	1.005 (1.000–1.011)	0.042
Single-lung ventilation time (min)	Per 1-unit increase	1.004 (0.997–1.011)	0.257
Blood loss (mL)	Per 1-unit increase	1.003 (0.998–1.007)	0.240
Minimally invasive approach	Yes vs No	0.976 (0.610–1.561)	0.920
Inflammatory and nutritional biomarkers			
Albumin (g/L)	Per 1-unit increase	0.999 (0.945–1.056)	0.970
CRP (mg/L)	Per 1-unit increase	1.099 (1.049–1.150)	<0.001
CALLY index	Per 1-unit increase	0.902 (0.863–0.944)	<0.001
AISI	Per 100-unit increase	1.264 (1.149–1.390)	<0.001

Data are presented as odds ratios (ORs) with 95% confidence intervals (CIs). Continuous variables were modeled per 1-unit increase unless otherwise specified. AISI was modeled per 100-unit increase to improve interpretability. For categorical variables with more than two levels, the lowest category served as the reference group.

**Table 3:** Multivariable logistic regression models for PPCs.

Variable	Model 1 clinical		Model 2 clinical + AISI		Model 3 clinical + CALLY		Model 4 clinical + AISI + CALLY	
	aOR (95% CI)	P value	aOR (95% CI)	P value	aOR (95% CI)	P value	aOR (95% CI)	P value
Clinical covariates included in all models								
Age (years)	1.038 (0.997-1.081)	0.070	1.029 (0.987-1.073)	0.176	1.036 (0.993-1.080)	0.102	1.029 (0.986-1.074)	0.186
Smoking history	1.402 (0.913-2.153)	0.123	1.355 (0.869-2.113)	0.180	1.330 (0.856-2.066)	0.204	1.308 (0.834-2.052)	0.243
COPD	1.743 (0.816-3.722)	0.151	1.707 (0.784-3.720)	0.178	1.589 (0.731-3.457)	0.243	1.600 (0.726-3.525)	0.244
ASA class (per category increase)	0.812 (0.551-1.195)	0.291	0.823 (0.553-1.223)	0.335	0.714 (0.478-1.065)	0.099	0.744 (0.495-1.116)	0.153
FEV1/FVC (%)	0.981 (0.964-0.997)	0.024	0.979 (0.962-0.997)	0.021	0.981 (0.964-0.998)	0.032	0.980 (0.963-0.998)	0.029
Operative duration (min)	1.007 (1.001-1.012)	0.019	1.006 (1.000-1.012)	0.035	1.006 (1.000-1.012)	0.034	1.006 (1.000-1.012)	0.044
Blood loss (mL)	1.003 (0.999-1.008)	0.147	1.004 (0.999-1.009)	0.107	1.003 (0.999-1.008)	0.173	1.004 (0.999-1.009)	0.131
Biomarkers added to the base clinical model								
AISI (per 100 units)	—	—	1.257 (1.141-1.384)	<0.001	—	—	1.195 (1.081-1.321)	<0.001
CALLY (per unit)	—	—	—	—	0.903 (0.862-0.945)	<0.001	0.926 (0.884-0.970)	0.001

Data are presented as adjusted odds ratios (aORs) with 95% confidence intervals (CIs). Model 1 included clinical covariates only. Model 2 included the clinical model plus AISI. Model 3 included the clinical model plus CALLY. Model 4 included the clinical model plus both AISI and CALLY. AISI was modeled per 100-unit increase, and CALLY was modeled per 1-unit increase.

### 3.4 Construction of the combined AISI-CALLY model

Because both AISI and CALLY remained independently associated with PPCs in multivariable analysis, a biomarker-based combined AISI-CALLY model was constructed using binary logistic regression. In this model, PPCs were entered as the dependent variable and AISI and CALLY as the independent predictors. When AISI was modeled per 100-unit increase and CALLY per 1-unit increase, the model was specified as follows:


logit(PPC)=−0.501+0.183×(AISI/100)−0.077×CALLY

Accordingly, each 100-unit increase in AISI was associated with increased odds of PPCs (OR 1.201, 95% CI 1.089–1.324, P < 0.001), whereas each 1-unit increase in CALLY was associated with decreased odds of PPCs (OR 0.926, 95% CI 0.885–0.969, P = 0.001). For each patient, the predicted probability generated from this model was used as the continuous prediction score in subsequent receiver operating characteristic (ROC), calibration, and decision-curve analyses. In parallel, an integrated final model was also developed by incorporating both biomarkers into the baseline clinical model.

### 3.5 Predictive performance of AISI, CALLY, and the combined model

**Figure 2:**
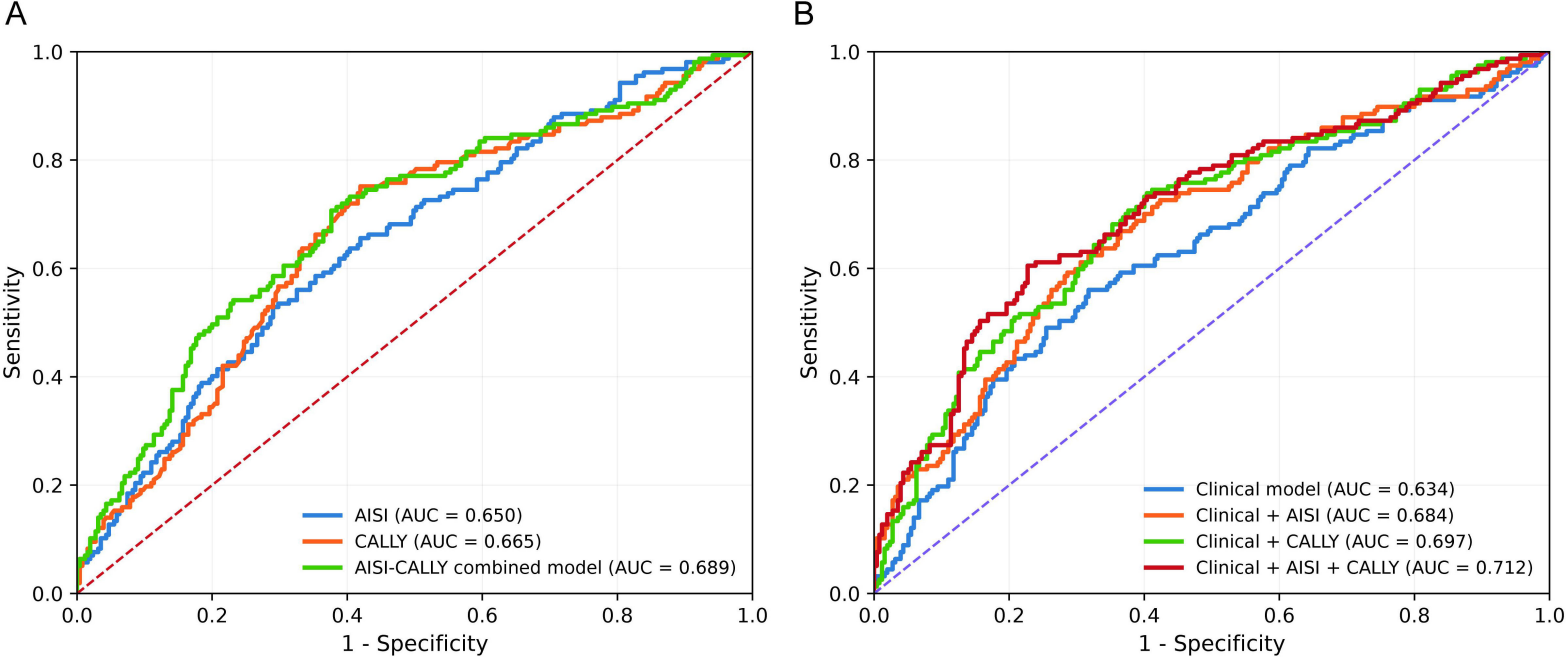
Receiver operating characteristic curves for biomarker-based and integrated models predicting PPCs. **(A)** Receiver operating characteristic (ROC) curves for biomarker-based models, including AISI alone, CALLY alone, and the combined AISI-CALLY model. **(B)** ROC curves for the baseline clinical model and integrated models incorporating AISI and/or CALLY. Area under the curve (AUC) values are shown for each model. The combined AISI-CALLY model was evaluated using model-predicted probabilities derived from logistic regression.

The predictive performances of AISI, CALLY, and their combined model are summarized in **Table 4** and **Figure 2**. Among the biomarker-based models, the AISI-CALLY combined model achieved the highest discrimination, with an AUC of 0.689 (95% CI 0.635–0.742), compared with 0.650 (95% CI 0.597–0.702) for AISI alone and 0.665 (95% CI 0.612–0.717) for CALLY alone. DeLong testing showed that the combined model significantly outperformed AISI alone (P = 0.027), whereas the difference between the combined model and CALLY alone did not reach statistical significance (P = 0.161) For the biomarker-based models, the combined AISI-CALLY model showed a sensitivity of 0.707, specificity of 0.624, accuracy of 0.655, and a Youden index of 0.331, with an optimal cutoff value of 0.367. In comparison, AISI alone showed higher specificity (0.710) but lower sensitivity (0.529), whereas CALLY alone showed higher sensitivity (0.752) but lower specificity (0.580). For the integrated clinical models, the baseline clinical model yielded an AUC of 0.634 (95% CI 0.581–0.689). Addition of AISI increased the AUC to 0.683 (95% CI 0.633–0.734; P = 0.027 vs clinical model), whereas addition of CALLY increased the AUC to 0.697 (95% CI 0.646–0.748; P = 0.007 vs clinical model). The final integrated model incorporating both AISI and CALLY achieved the highest AUC of 0.712 (95% CI 0.658–0.762), with a sensitivity of 0.605, specificity of 0.761, accuracy of 0.701, and a Youden index of 0.366. DeLong testing showed that this final model significantly outperformed the baseline clinical model (P = 0.003) and showed borderline improvement over the clinical model plus AISI (P = 0.050), whereas the difference compared with the clinical model plus CALLY was not statistically significant (P = 0.246). Taken together, these findings indicate that joint consideration of AISI and CALLY modestly improved discrimination compared with AISI alone and yielded the highest, albeit still moderate, predictive performance when integrated with clinical variables.

**Table 4:** Predictive performance of AISI, CALLY, and combined models for PPCs.

Model	AUC	95% CI	Optimal cutoff	Sensitivity	Specificity	PPV	NPV	Accuracy	Youden index	DeLong comparison	P value
Biomarker-based models											
AISI alone	0.650	0.597–0.702	0.388	0.529	0.710	0.529	0.710	0.641	0.238	Reference	—
CALLY alone	0.665	0.612–0.717	0.391	0.752	0.580	0.524	0.791	0.646	0.332	Reference	—
AISI-CALLY combined model	0.689	0.635–0.742	0.367	0.707	0.624	0.536	0.776	0.655	0.331	vs AISI alone; vs CALLY alone	0.027; 0.161
Integrated clinical models											
Clinical model	0.634	0.581–0.689	0.413	0.516	0.725	0.536	0.709	0.646	0.241	Reference	—
Clinical + AISI	0.683	0.633–0.734	0.389	0.618	0.702	0.561	0.749	0.670	0.320	vs Clinical model	0.027
Clinical + CALLY	0.697	0.646–0.748	0.382	0.694	0.647	0.548	0.775	0.665	0.341	vs Clinical model	0.007
Clinical + AISI + CALLY	0.712	0.658–0.762	0.432	0.605	0.761	0.609	0.758	0.701	0.366	vs Clinical model; vs Clinical + AISI; vs Clinical + CALLY	0.003; 0.050; 0.246

AUC indicates area under the receiver operating characteristic curve; CI, confidence interval; PPV, positive predictive value; NPV, negative predictive value. The AISI-CALLY combined model was constructed using binary logistic regression with PPCs as the dependent variable and AISI and CALLY as predictors; model performance was evaluated based on the predicted probabilities derived from the model. The integrated final model included the clinical model plus both AISI and CALLY. P values for DeLong comparisons refer to comparisons with the specified reference model(s).

### 3.6 Calibration and clinical utility of the final model

**Figure 3:**
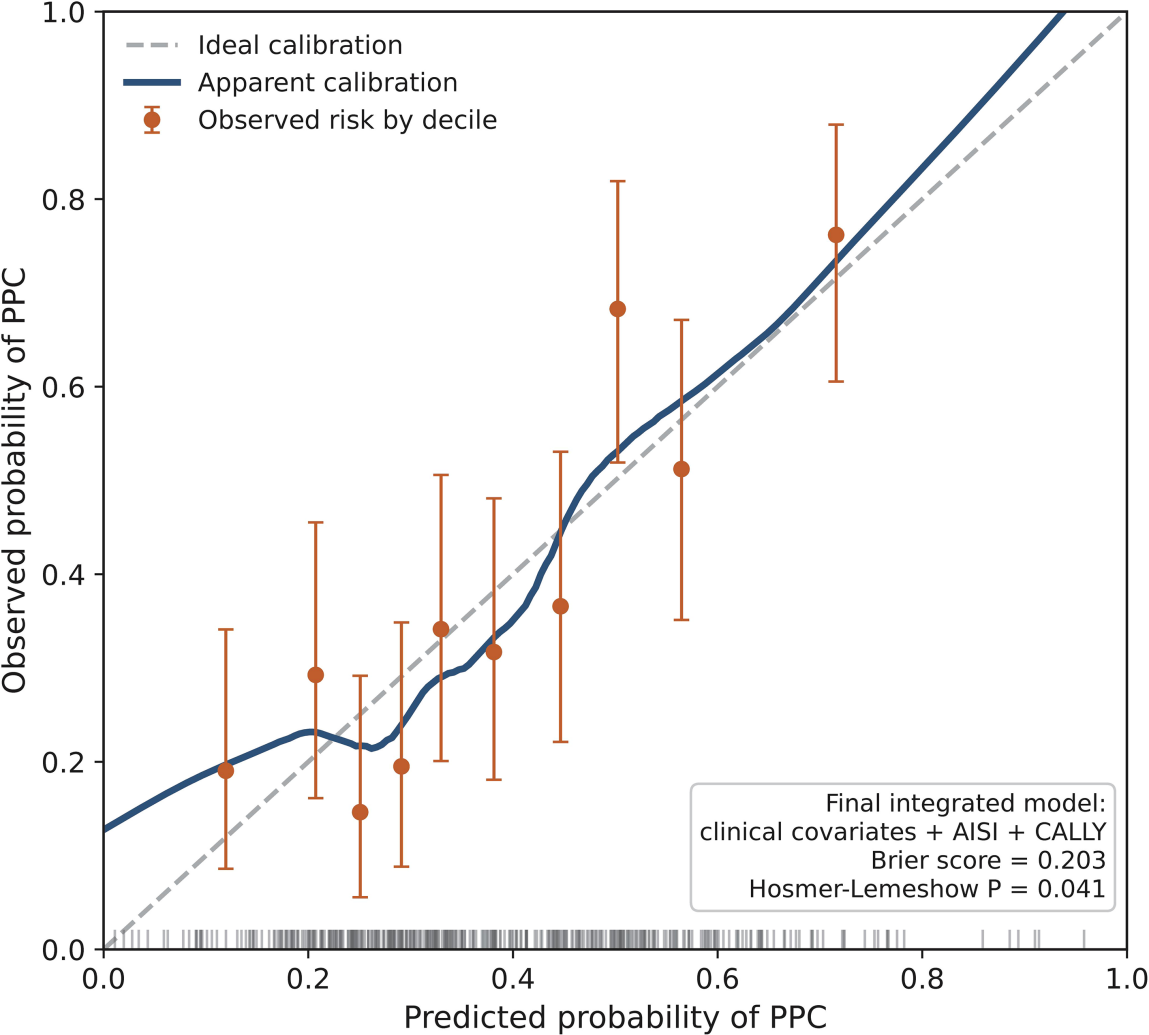
Calibration curve of the final integrated model for PPC prediction. Calibration plot for the final integrated model incorporating clinical variables, AISI, and CALLY. The dashed diagonal line indicates ideal calibration, the solid line indicates apparent calibration, and the points represent observed PPC rates by decile of predicted risk with corresponding error bars. Model calibration was additionally assessed using the Brier score and the Hosmer-Lemeshow goodness-of-fit test.

**Figure 4:**
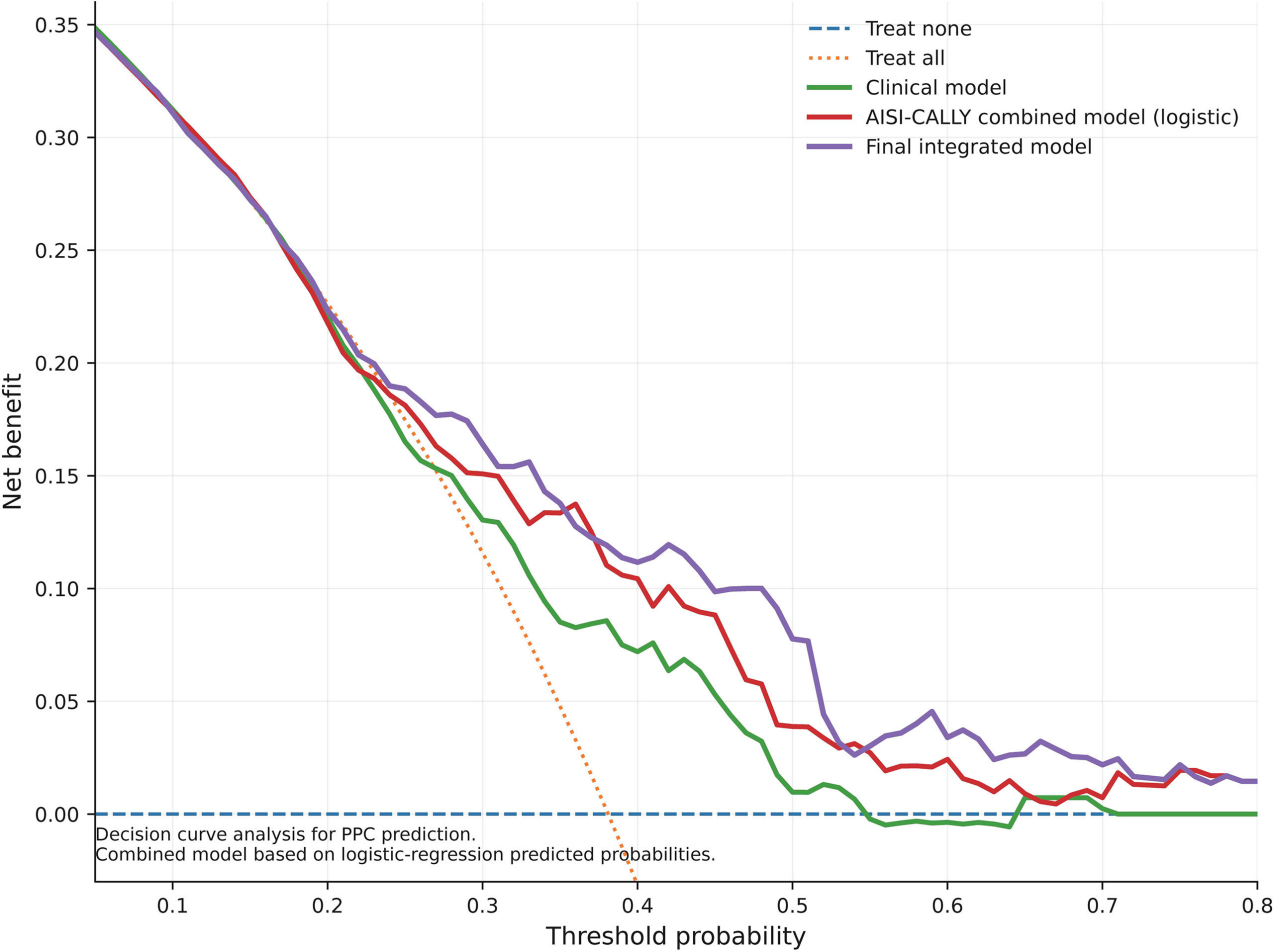
Decision curve analysis of prediction models for PPCs. Decision curve analysis comparing the baseline clinical model, the combined AISI-CALLY model, and the final integrated model for prediction of PPCs. Net benefit is plotted across a range of threshold probabilities. The “treat all” and “treat none” strategies are shown as reference lines. Higher net benefit across clinically relevant threshold probabilities indicates greater potential clinical usefulness.

The calibration performance of the final integrated model is shown in **Figure 3**. Overall, the model demonstrated reasonable agreement between predicted and observed risks of PPCs, with a Brier score of 0.203. The Hosmer-Lemeshow goodness-of-fit test yielded a P value of 0.041, indicating that some deviation between predicted and observed event rates remained across risk strata. The clinical utility of the prediction models was further evaluated using decision curve analysis (**Figure 4**). Across a clinically relevant range of threshold probabilities, both the biomarker-based combined AISI-CALLY model and the final integrated model provided greater net benefit than the “treat all” and “treat none” strategies. The final integrated model showed the most favorable overall net benefit profile and generally outperformed the baseline clinical model, indicating that incorporation of AISI and CALLY may improve the clinical usefulness of PPC risk stratification beyond conventional clinical variables alone. Taken together, these findings suggest that the final integrated model achieved the highest discriminative performance among the evaluated models and demonstrated reasonable calibration and potential clinical utility for preoperative PPC risk assessment.

### 3.7 Sensitivity analysis using propensity score matching

To further assess the robustness of the primary findings, propensity score matching (PSM) was performed as a sensitivity analysis. After 1:1 nearest-neighbor matching without replacement, 134 matched pairs were obtained. Baseline characteristics before and after matching are summarized in **Supplementary Table 1**, and covariate balance is illustrated in **Supplementary Figure 2**. Overall, baseline balance was substantially improved after matching, although AISI and CALLY remained different between groups because these biomarkers were not included as matching variables and were intentionally retained for post-matching association analyses. Multivariable logistic regression analyses in the matched cohort are presented in **Supplementary Table 2**. In the matched clinical model plus AISI, AISI remained independently associated with PPCs (aOR 1.229 per 100 units, 95% CI 1.092–1.383, P < 0.001). In the matched clinical model plus CALLY, CALLY also remained independently associated with PPCs (aOR 0.923 per unit, 95% CI 0.878–0.970, P = 0.002). Importantly, in the fully adjusted matched-cohort model including both biomarkers, both AISI (aOR 1.179, 95% CI 1.046–1.330, P = 0.007) and CALLY (aOR 0.942, 95% CI 0.896–0.991, P = 0.021) retained statistical significance. Receiver operating characteristic analyses in the matched cohort are shown in **Supplementary Figure 3**. The AUCs were 0.622 for AISI alone, 0.643 for CALLY alone, and 0.638 for the combined AISI-CALLY model. Although the overall discrimination was modest after matching, the direction of association remained consistent with that observed in the full cohort. Taken together, these matched-cohort analyses support the overall consistency of the primary findings and indicate that the independent and complementary associations of AISI and CALLY with PPCs were not solely attributable to baseline imbalances in the unmatched cohort.

## 4 Discussion

In this retrospective cohort of patients with ESCC undergoing McKeown esophagectomy after nICT, preoperative AISI and CALLY were independently associated with PPCs and provided complementary prognostic information. Their joint incorporation improved discrimination beyond a baseline clinical model, but the magnitude of improvement was modest. Accordingly, the main contribution of these biomarkers is not the creation of a high-accuracy standalone prediction tool, but the refinement of preoperative pulmonary risk assessment through biologically informed, readily available adjunctive markers.

Several points deserve emphasis. First, the burden of PPCs remained substantial in this cohort, affecting more than one-third of patients (24). This finding reinforces that pulmonary morbidity continues to be a central perioperative challenge after esophagectomy, even in the contemporary setting of multimodal treatment (25). In the context of nICT, this issue becomes more clinically relevant because perioperative risk is shaped not only by surgical trauma and baseline lung function, but also by treatment-related alterations in systemic inflammation, immune homeostasis, and nutritional reserve (26, 27). The present findings therefore support the premise that conventional clinical variables alone incompletely capture host vulnerability in patients proceeding to McKeown esophagectomy after systemic neoadjuvant therapy.

The observed association between higher AISI and increased PPC risk is biologically plausible. AISI integrates neutrophils, platelets, monocytes, and lymphocytes into a composite measure of systemic inflammatory burden and dysregulated innate-adaptive immune balance (28). In the perioperative setting, such a profile can be interpreted as a marker of a host state characterized by heightened inflammatory activation and reduced immunologic resilience (29). This may contribute to pulmonary endothelial injury, microcirculatory dysfunction, exaggerated postoperative inflammatory responses, and impaired control of infectious or aspiration-related insults (30, 31). These mechanisms are especially relevant after McKeown esophagectomy, where one-lung ventilation, thoracic manipulation, airway vulnerability, and postoperative secretion retention may already predispose patients to pulmonary injury. In this setting, elevated AISI is best understood not as an isolated hematologic abnormality, but as a surrogate of systemic biological stress before major thoracic surgery.

The inverse association between CALLY and PPCs complements this interpretation (32). Compared with cell-count-based inflammatory indices, CALLY integrates C-reactive protein, albumin, and lymphocyte count, thereby capturing acute-phase inflammation, nutritional reserve, and immune competence within a single measure. This multidimensionality is important because PPCs after esophagectomy are not solely inflammatory events (17, 33). They also reflect the patient’s capacity to tolerate operative stress, maintain barrier function, support tissue recovery, and mount effective host defense during the early postoperative period. A lower CALLY therefore likely identifies patients with combined inflammatory activation, impaired protein reserve, and weakened immune status, all of which are unfavorable for pulmonary recovery after nICT and major thoracic surgery. In this respect, the present findings are directionally consistent with prior work suggesting that immunonutritional markers are clinically relevant in esophageal cancer, while extending that concept specifically to composite PPC risk after nICT-treated McKeown esophagectomy (34, 35).

A key result of this study is that AISI and CALLY retained statistical significance in the same multivariable model despite their moderate inverse correlation. This finding has both methodological and biological implications. Methodologically, the low collinearity metrics indicate that the two indices are not interchangeable surrogates and can be evaluated jointly without major model instability. Biologically, the result suggests that systemic inflammatory burden and integrated inflammation-nutrition-immune status represent related but nonredundant dimensions of perioperative vulnerability (36, 37). This distinction helps explain why the combined biomarker model performed better than AISI alone and why the best overall discrimination was achieved when both biomarkers were added to the baseline clinical model. At the same time, the pattern of model comparisons is equally informative: the combined biomarker model was significantly better than AISI alone, but not significantly better than CALLY alone, and the final integrated model did not significantly outperform the clinical model plus CALLY. This suggests that CALLY already captures a broader component of biologic risk, whereas AISI provides incremental but partly overlapping information. In other words, the two markers are complementary, but not additive in a fully independent manner. This point is central to the interpretation of the study. The biomarker-only AISI-CALLY model achieved only moderate discrimination, and even the final integrated model reached an AUC of 0.712 rather than the level usually associated with a high-performance clinical prediction tool. For that reason, the present findings should not be overinterpreted as evidence that preoperative biomarker assessment alone can accurately classify individual patients. A more appropriate conclusion is that AISI and CALLY improve risk estimation at the margin by adding biologically relevant information to conventional perioperative assessment. This is also consistent with the balance of sensitivity and specificity observed across models. AISI alone favored specificity at the expense of sensitivity, whereas CALLY alone favored sensitivity at the expense of specificity; the combined model partially balanced these trade-offs, and the integrated model achieved the best overall operating characteristics (38).

Such a pattern is clinically plausible because different biomarkers often capture different components of susceptibility rather than functioning as mutually substitutable predictors (39). The calibration and decision-curve findings support this restrained interpretation. The final integrated model showed the best net benefit profile across clinically relevant threshold probabilities, indicating that the combined use of clinical variables, AISI, and CALLY has potential utility for preoperative risk stratification. However, calibration was not fully satisfactory, as reflected by a Brier score of 0.203 and a Hosmer-Lemeshow P value of 0.041. Thus, although the model demonstrated useful signal, it still requires refinement before any broader clinical implementation. For a single-center retrospective model developed in a relatively specific treatment context, this is not unexpected. It instead underscores that discrimination and clinical utility can be promising while calibration remains imperfect, and that external recalibration is a necessary next step rather than an afterthought. Importantly, the ROC results in **Figure 2** should be interpreted with caution. Although the AISI-CALLY combined model and the final integrated model showed better discrimination than some comparator models, the absolute AUC values remained only moderate (0.689 for the biomarker-only combined model and 0.712 for the final integrated model). These findings do not support regarding the model as a high-accuracy stand-alone prediction tool. Therefore, if the predictive utility were overstated, the conclusion could indeed be biased. In the present study, however, the main conclusion is not that AISI and CALLY alone can precisely classify individual patients, but that they are independently associated with PPCs and provide modest incremental predictive information beyond conventional clinical variables. This interpretation is supported by the multivariable regression analyses, in which both biomarkers remained statistically significant after adjustment, whereas the ROC analysis mainly informs the magnitude of discrimination rather than the existence of association. The matched-cohort analysis adds further nuance. After PSM, both AISI and CALLY remained independently associated with PPCs, which supports the robustness of the primary associations and argues against a purely confounding-driven explanation. However, discrimination in the matched cohort was attenuated, and the combined biomarker model no longer showed a clear advantage.

This attenuation should not be viewed as contradictory to the main findings. Matching reduces baseline heterogeneity and narrows the distribution of clinical risk, which commonly decreases the apparent performance of prediction models. The matched analysis therefore supports the stability of the associations, while also showing that the predictive gain of the biomarker combination is context-dependent and weaker than might be inferred from the unmatched cohort alone (40). This is precisely why the present study is stronger when framed as an analysis of association plus modest incremental value, rather than as derivation of a high-performance prediction model.

The study also has several clinical implications. Because AISI and CALLY are derived from routine preoperative laboratory tests, they are inexpensive, objective, and readily deployable within ordinary perioperative workflows. Their practical value lies in identifying patients whose biological reserve appears less favorable even when conventional demographic or tumor-related characteristics are not markedly different (41). In principle, such patients could be considered for more intensive respiratory prehabilitation, closer surveillance for early pulmonary deterioration, stricter perioperative pulmonary hygiene, or heightened caution in postoperative monitoring. At the same time, the present data do not support deterministic decision-making based on these biomarkers alone, because their discriminative performance remained only moderate rather than high (42, 43). Their appropriate role is as accessible adjuncts that help refine risk stratification, particularly when interpreted alongside pulmonary function, operative burden, and overall clinical condition.

Several limitations should be acknowledged. First, this was a retrospective single-center study, and residual confounding cannot be fully excluded despite multivariable adjustment and propensity score matching. Second, the biomarkers were assessed at a single preoperative time point. Dynamic changes during nICT, recovery after the last treatment cycle, or very early perioperative trajectories were not evaluated and may contain additional prognostic information. Third, PPCs were analyzed as a composite endpoint. This improves clinical relevance and statistical efficiency, but it may obscure whether AISI and CALLY relate more strongly to some pulmonary events than to others. Fourth, although the cohort size is meaningful for this specific clinical scenario, it remains limited for developing and calibrating a broadly transportable prediction model. Fifth, the final model demonstrated only moderate discrimination and imperfect calibration, so it should not be interpreted as a highly accurate clinical prediction tool or as ready for direct clinical deployment. If the predictive performance were overstated, this could bias the conclusion. Accordingly, our findings should be interpreted as showing modest incremental risk-stratification value rather than definitive stand-alone predictive accuracy. Finally, compared with earlier exploratory work centered on different biomarker combinations, the present analysis shifted its focus to AISI and CALLY in an expanded cohort. This change improved alignment with the biology of inflammation-related perioperative vulnerability, but it also means that the current study should be interpreted as a refined evaluation of a different biomarker framework rather than a direct one-to-one validation of earlier models.

In summary, preoperative AISI and CALLY captured distinct but complementary dimensions of host susceptibility to PPCs after McKeown esophagectomy following nICT. Their combined use provided only modest incremental predictive value beyond standard clinical assessment and should therefore be regarded as an accessible adjunct for preoperative pulmonary risk stratification rather than a stand-alone prediction tool. Future studies should focus on prospective multicenter validation, external recalibration, and the integration of dynamic treatment-related and perioperative variables to determine whether biologically informed risk assessment can be translated into more personalized perioperative management.

## Data Availability

The original contributions presented in the study are included in the article/**Supplementary Material**. Further inquiries can be directed to the corresponding authors.
