# Supplementary figures and images for: Association and incremental predictive value of preoperative AISI and CALLY for postoperative pulmonary complications after McKeown esophagectomy following neoadjuvant chemoimmunotherapy
Source: Front Immunol. 2026 Apr 15;17:1642365. doi: 10.3389/fimmu.2026.1642365 (PMC13124699; doi:10.3389/fimmu.2026.1642365)

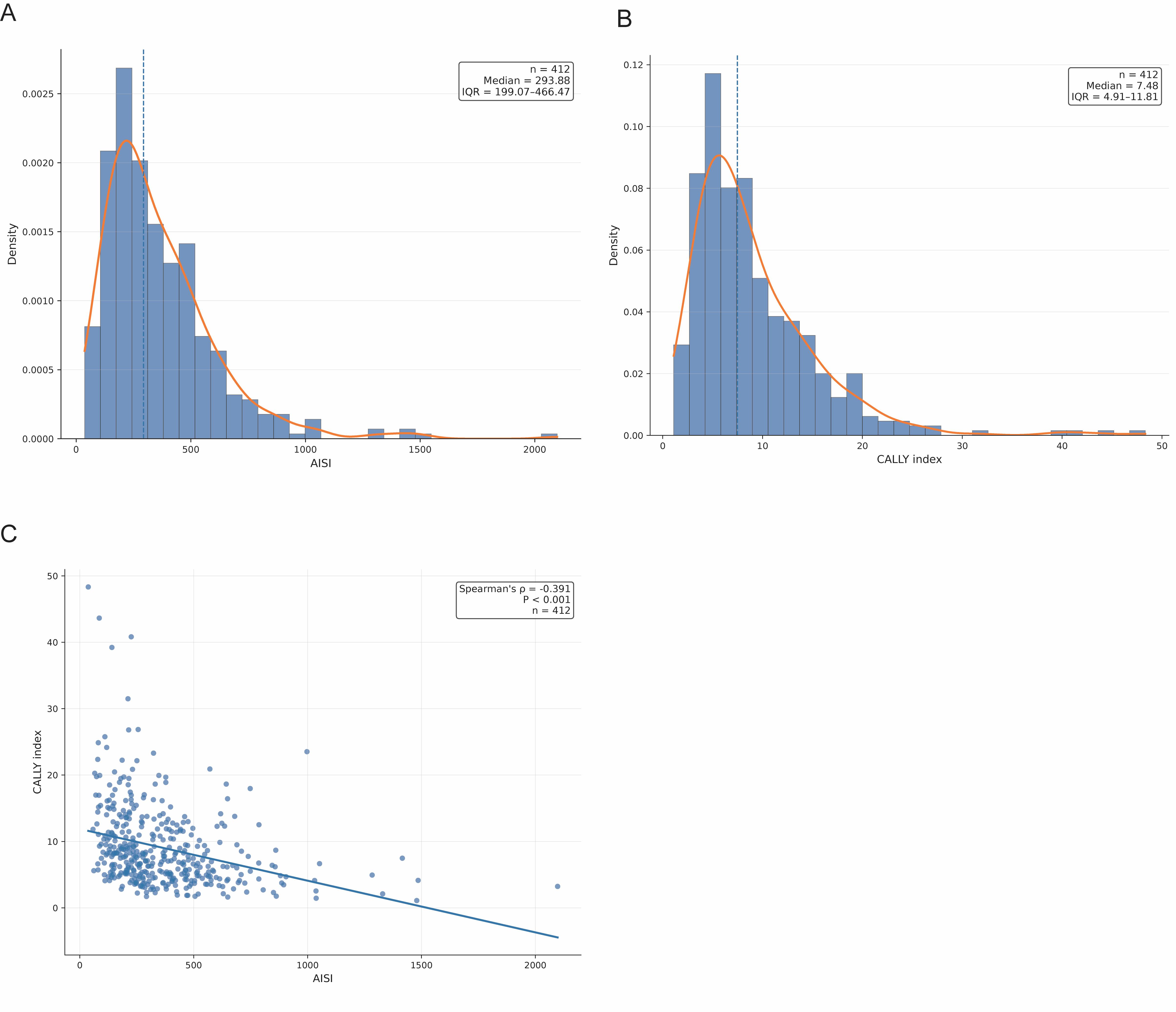

Supplement: Supplementary file 1 [file Image1.jpeg]

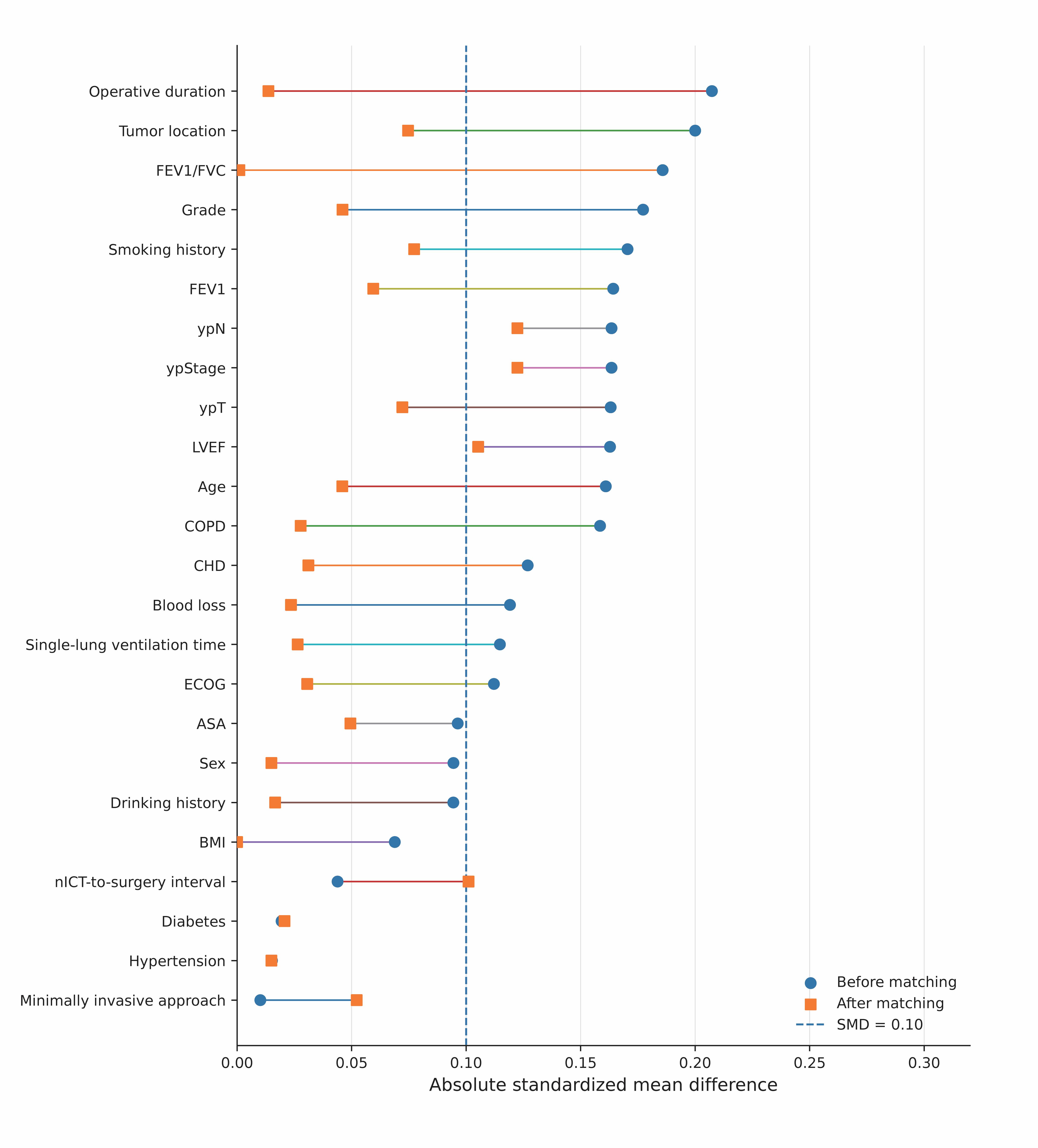

Supplement: Supplementary file 2 [file Image2.jpeg]

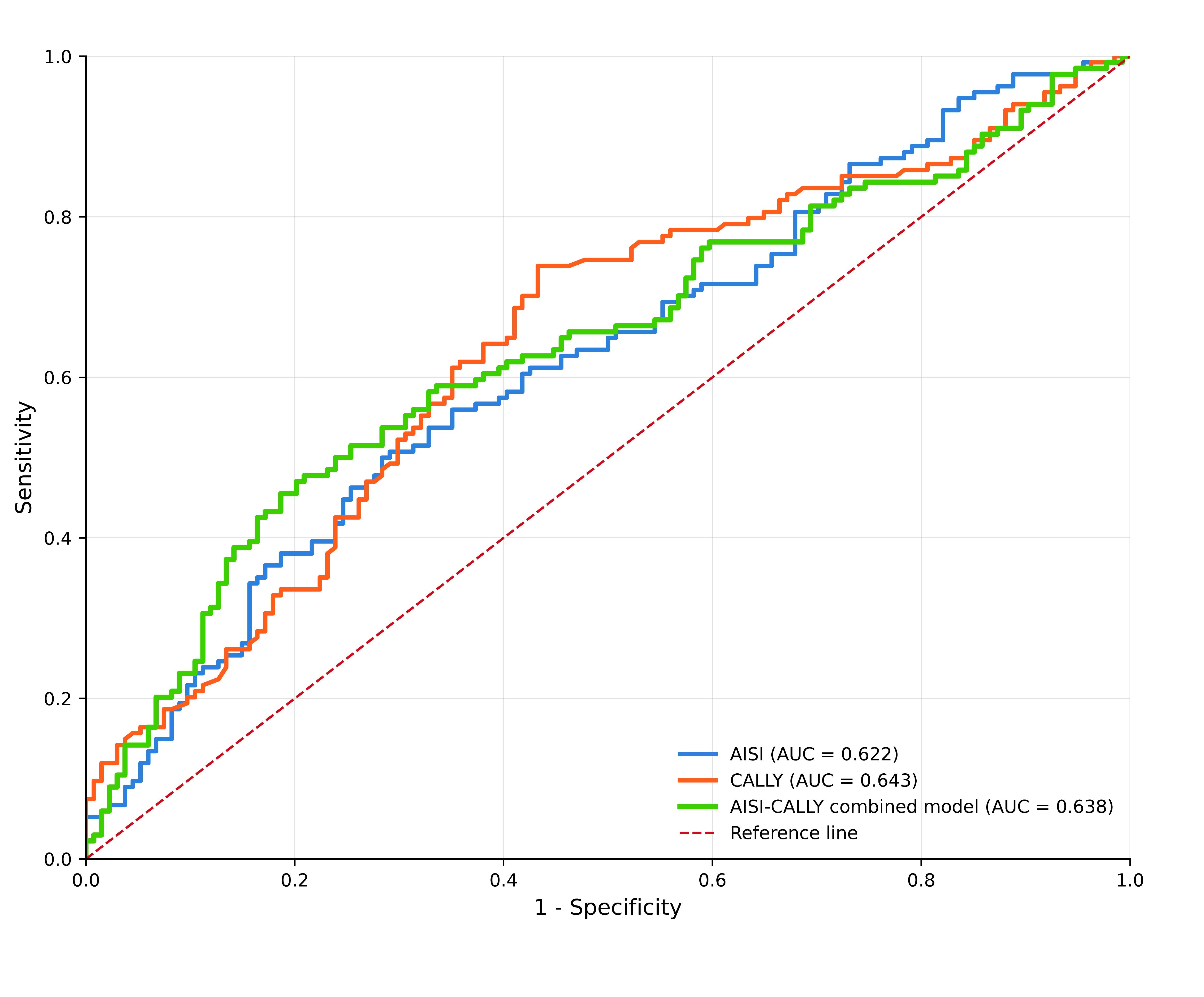

Supplement: Supplementary file 3 [file Image3.jpeg]
