# Supplementary material for: Association and incremental predictive value of preoperative AISI and CALLY for postoperative pulmonary complications after McKeown esophagectomy following neoadjuvant chemoimmunotherapy
Source: Front Immunol. 2026 Apr 15;17:1642365. doi: 10.3389/fimmu.2026.1642365 (PMC13124699; doi:10.3389/fimmu.2026.1642365)
Supplement: Supplementary file 4 [file Supplementaryfile1.docx]

Supplementary Table 1. Baseline characteristics before and after propensity score matching

| Variable | Before matching | | | | After matching | | | |
| --- | --- | --- | --- | --- | --- | --- | --- | --- |
|  | No PPC | PPC | P value | SMD | No PPC | PPC | P value | SMD |
| Continuous variables | | | | | | | | |
| Age, years | 67.89 ± 5.15 | 68.71 ± 5.02 | 0.112 | 0.161 | 68.59 ± 5.24 | 68.36 ± 5.03 | 0.707 | 0.046 |
| BMI, kg/m² | 22.81 ± 2.26 | 22.98 ± 2.59 | 0.504 | 0.069 | 22.98 ± 2.44 | 22.98 ± 2.45 | 1.000 | 0.000 |
| FEV1, L | 2.14 ± 0.42 | 2.21 ± 0.41 | 0.105 | 0.164 | 2.22 ± 0.42 | 2.19 ± 0.41 | 0.627 | 0.059 |
| FEV1/FVC, % | 104.77 ± 11.98 | 102.51 ± 12.33 | 0.069 | 0.186 | 103.27 ± 11.63 | 103.26 ± 12.02 | 0.993 | 0.001 |
| LVEF, % | 66.24 ± 2.26 | 65.87 ± 2.35 | 0.111 | 0.163 | 65.69 ± 2.29 | 65.94 ± 2.36 | 0.390 | 0.105 |
| nICT-to-surgery interval, days | 30.75 ± 7.26 | 30.43 ± 7.61 | 0.667 | 0.044 | 29.81 ± 7.34 | 30.56 ± 7.58 | 0.409 | 0.101 |
| Operative duration, min | 272.08 ± 37.77 | 280.00 ± 38.60 | 0.042 | 0.207 | 277.47 ± 36.26 | 277.99 ± 38.91 | 0.911 | 0.014 |
| Single-lung ventilation time, min | 145.02 ± 28.26 | 148.30 ± 28.97 | 0.260 | 0.115 | 146.99 ± 28.55 | 147.74 ± 27.89 | 0.829 | 0.026 |
| Blood loss, mL | 128.71 ± 44.51 | 134.04 ± 44.99 | 0.242 | 0.119 | 132.78 ± 45.15 | 131.75 ± 42.32 | 0.847 | 0.024 |
| Albumin, g/L | 39.65 ± 3.57 | 39.64 ± 3.68 | 0.971 | 0.004 | 39.38 ± 3.79 | 39.86 ± 3.74 | 0.301 | 0.127 |
| CRP, mg/L | 6.51 (4.45-9.16) | 8.32 (5.85-11.66) | <0.001 | 0.423 | 6.86 (4.71-9.09) | 8.32 (5.62-11.68) | 0.004 | 0.407 |
| CALLY index | 8.68 (5.53-12.80) | 5.72 (4.28-8.06) | <0.001 | 0.497 | 8.35 (5.30-12.61) | 5.71 (4.14-8.46) | <0.001 | 0.402 |
| AISI | 260.06 (174.52-409.29) | 385.94 (228.00-548.28) | <0.001 | 0.517 | 277.06 (175.52-394.61) | 377.75 (222.44-513.72) | <0.001 | 0.444 |
| Categorical variables | | | | | | | | |
| Sex | Female: 105 (41.2%); Male: 150 (58.8%) | Female: 72 (45.9%); Male: 85 (54.1%) | 0.406 | 0.094 | Female: 62 (46.3%); Male: 72 (53.7%) | Female: 61 (45.5%); Male: 73 (54.5%) | 1.000 | 0.015 |
| Smoking history | No: 172 (67.5%); Yes: 83 (32.5%) | No: 93 (59.2%); Yes: 64 (40.8%) | 0.113 | 0.170 | No: 82 (61.2%); Yes: 52 (38.8%) | No: 87 (64.9%); Yes: 47 (35.1%) | 0.613 | 0.077 |
| Drinking history | No: 191 (74.9%); Yes: 64 (25.1%) | No: 111 (70.7%); Yes: 46 (29.3%) | 0.411 | 0.094 | No: 96 (71.6%); Yes: 38 (28.4%) | No: 97 (72.4%); Yes: 37 (27.6%) | 1.000 | 0.017 |
| Hypertension | No: 141 (55.3%); Yes: 114 (44.7%) | No: 88 (56.1%); Yes: 69 (43.9%) | 0.962 | 0.015 | No: 73 (54.5%); Yes: 61 (45.5%) | No: 74 (55.2%); Yes: 60 (44.8%) | 1.000 | 0.015 |
| Diabetes | No: 213 (83.5%); Yes: 42 (16.5%) | No: 130 (82.8%); Yes: 27 (17.2%) | 0.955 | 0.019 | No: 114 (85.1%); Yes: 20 (14.9%) | No: 113 (84.3%); Yes: 21 (15.7%) | 1.000 | 0.021 |
| Coronary heart disease | No: 173 (67.8%); Yes: 82 (32.2%) | No: 97 (61.8%); Yes: 60 (38.2%) | 0.250 | 0.127 | No: 87 (64.9%); Yes: 47 (35.1%) | No: 85 (63.4%); Yes: 49 (36.6%) | 0.899 | 0.031 |
| COPD | No: 240 (94.1%); Yes: 15 (5.9%) | No: 141 (89.8%); Yes: 16 (10.2%) | 0.156 | 0.158 | No: 124 (92.5%); Yes: 10 (7.5%) | No: 123 (91.8%); Yes: 11 (8.2%) | 1.000 | 0.028 |
| ASA class | 1: 67 (26.3%); 2: 171 (67.1%); 3: 17 (6.7%) | 1: 44 (28.0%); 2: 106 (67.5%); 3: 7 (4.5%) | 0.629 | 0.096 | 1: 37 (27.6%); 2: 89 (66.4%); 3: 8 (6.0%) | 1: 40 (29.9%); 2: 87 (64.9%); 3: 7 (5.2%) | 0.902 | 0.049 |
| ECOG | 0: 133 (52.2%); 1: 100 (39.2%); 2: 22 (8.6%) | 0: 83 (52.9%); 1: 65 (41.4%); 2: 9 (5.7%) | 0.547 | 0.112 | 0: 66 (49.3%); 1: 60 (44.8%); 2: 8 (6.0%) | 0: 67 (50.0%); 1: 58 (43.3%); 2: 9 (6.7%) | 0.951 | 0.031 |
| Tumor location | Upper: 64 (25.1%); Middle: 134 (52.5%); Low: 57 (22.4%) | Upper: 34 (21.7%); Middle: 74 (47.1%); Low: 49 (31.2%) | 0.135 | 0.200 | Upper: 29 (21.6%); Middle: 68 (50.7%); Low: 37 (27.6%) | Upper: 33 (24.6%); Middle: 63 (47.0%); Low: 38 (28.4%) | 0.794 | 0.075 |
| Tumor grade | Well: 154 (60.4%); Moderate: 82 (32.2%); Poor: 19 (7.5%) | Well: 81 (51.6%); Moderate: 63 (40.1%); Poor: 13 (8.3%) | 0.206 | 0.177 | Well: 71 (53.0%); Moderate: 53 (39.6%); Poor: 10 (7.5%) | Well: 73 (54.5%); Moderate: 50 (37.3%); Poor: 11 (8.2%) | 0.922 | 0.046 |
| ypT stage | T0: 74 (29.0%); T1: 118 (46.3%); T2: 51 (20.0%); T3: 12 (4.7%) | T0: 57 (36.3%); T1: 60 (38.2%); T2: 33 (21.0%); T3: 7 (4.5%) | 0.370 | 0.163 | T0: 48 (35.8%); T1: 55 (41.0%); T2: 26 (19.4%); T3: 5 (3.7%) | T0: 47 (35.1%); T1: 53 (39.6%); T2: 27 (20.1%); T3: 7 (5.2%) | 0.940 | 0.072 |
| ypN stage | N0: 203 (79.6%); N1: 38 (14.9%); N2: 7 (2.7%); N3: 7 (2.7%) | N0: 123 (78.3%); N1: 25 (15.9%); N2: 8 (5.1%); N3: 1 (0.6%) | 0.286 | 0.164 | N0: 107 (79.9%); N1: 22 (16.4%); N2: 4 (3.0%); N3: 1 (0.7%) | N0: 110 (82.1%); N1: 18 (13.4%); N2: 6 (4.5%) | 0.606 | 0.122 |
| ypTNM stage | 0: 63 (24.7%); I: 129 (50.6%); II: 56 (22.0%); III: 7 (2.7%) | 0: 47 (29.9%); I: 73 (46.5%); II: 36 (22.9%); III: 1 (0.6%) | 0.309 | 0.164 | 0: 39 (29.1%); I: 63 (47.0%); II: 31 (23.1%); III: 1 (0.7%) | 0: 42 (31.3%); I: 65 (48.5%); II: 27 (20.1%) | 0.701 | 0.122 |
| Minimally invasive approach | No: 59 (23.1%); Yes: 196 (76.9%) | No: 37 (23.6%); Yes: 120 (76.4%) | 1.000 | 0.010 | No: 34 (25.4%); Yes: 100 (74.6%) | No: 31 (23.1%); Yes: 103 (76.9%) | 0.776 | 0.052 |

Note: Before matching, the non-PPC and PPC groups included 255 and 157 patients, respectively. After 1:1 propensity score matching, 134 matched pairs were obtained. Data are presented as mean ± SD, median (IQR), or n (%), as appropriate. SMD indicates standardized mean difference.
