# Supplementary material for: Association and incremental predictive value of preoperative AISI and CALLY for postoperative pulmonary complications after McKeown esophagectomy following neoadjuvant chemoimmunotherapy
Source: Front Immunol. 2026 Apr 15;17:1642365. doi: 10.3389/fimmu.2026.1642365 (PMC13124699; doi:10.3389/fimmu.2026.1642365)
Supplement: Supplementary file 5 [file Supplementaryfile2.docx]

Supplementary Table 2. Multivariable logistic regression analyses for PPCs in the propensity score-matched cohort

| Variable | Model 1  Clinical | | Model 2  Clinical + AISI | | Model 3  Clinical + CALLY | | Model 4  Clinical + AISI + CALLY | |
| --- | --- | --- | --- | --- | --- | --- | --- | --- |
|  | aOR (95% CI) | P value | aOR (95% CI) | P value | aOR (95% CI) | P value | aOR (95% CI) | P value |
| **Clinical covariates included in all matched-cohort models** | | | | | | | | |
| Age (years) | 0.989 (0.943-1.037) | 0.647 | 0.984 (0.937-1.034) | 0.527 | 0.987 (0.940-1.037) | 0.599 | 0.984 (0.936-1.034) | 0.521 |
| Smoking history | 0.837 (0.505-1.385) | 0.488 | 0.794 (0.472-1.337) | 0.386 | 0.820 (0.490-1.373) | 0.451 | 0.790 (0.467-1.337) | 0.380 |
| COPD | 1.104 (0.447-2.726) | 0.830 | 1.065 (0.422-2.685) | 0.894 | 1.044 (0.414-2.635) | 0.927 | 1.031 (0.404-2.632) | 0.949 |
| ASA class | 0.904 (0.575-1.419) | 0.660 | 0.905 (0.570-1.438) | 0.674 | 0.839 (0.529-1.333) | 0.458 | 0.858 (0.537-1.372) | 0.522 |
| FEV1/FVC (%) | 1.000 (0.980-1.021) | 0.964 | 0.998 (0.977-1.020) | 0.870 | 1.001 (0.980-1.022) | 0.950 | 0.999 (0.978-1.021) | 0.929 |
| Operative duration (min) | 1.001 (0.994-1.007) | 0.880 | 1.000 (0.993-1.007) | 0.972 | 1.000 (0.993-1.007) | 0.968 | 1.000 (0.993-1.006) | 0.893 |
| Blood loss (mL) | 0.999 (0.994-1.005) | 0.798 | 1.000 (0.994-1.006) | 0.941 | 0.999 (0.994-1.005) | 0.803 | 1.000 (0.994-1.006) | 0.922 |
| **Biomarkers added to the matched-cohort clinical model** | | | | | | | | |
| AISI (per 100 units) | — | — | 1.229 (1.092-1.383) | <0.001 | — | — | 1.179 (1.046-1.330) | 0.007 |
| CALLY (per 1 unit) | — | — | — | — | 0.923 (0.878-0.970) | 0.002 | 0.942 (0.896-0.991) | 0.021 |

Note: Data are presented as adjusted odds ratios (aORs) with 95% confidence intervals (CIs). Model 1 included matched-cohort clinical covariates only. Model 2 included the matched clinical model plus AISI. Model 3 included the matched clinical model plus CALLY. Model 4 included the matched clinical model plus both AISI and CALLY. AISI was modeled per 100-unit increase, and CALLY was modeled per 1-unit increase.
